# Functional characterization of *Arabidopsis thaliana *transthyretin-like protein

**DOI:** 10.1186/1471-2229-10-30

**Published:** 2010-02-18

**Authors:** João Pessoa, Zsuzsa Sárkány, Frederico Ferreira-da-Silva, Sónia Martins, Maria R Almeida, Jianming Li, Ana M Damas

**Affiliations:** 1 IBMC - Instituto de Biologia Molecular e Celular, Universidade do Porto, Rua do Campo Alegre 823, 4150-180 Porto, Portugal; 2 ICBAS - Instituto de Ciências Biomédicas de Abel Salazar, Universidade do Porto, Largo Prof. Abel Salazar 2, 4099-003 Porto, Portugal; 3 Department of Molecular, Cellular, and Developmental Biology, University of Michigan, Ann Arbor, Michigan 48109-1048, USA

## Abstract

**Background:**

*Arabidopsis thaliana *transthyretin-like (TTL) protein is a potential substrate in the brassinosteroid signalling cascade, having a role that moderates plant growth. Moreover, sequence homology revealed two sequence domains similar to 2-oxo-4-hydroxy-4-carboxy-5-ureidoimidazoline (OHCU) decarboxylase (N-terminal domain) and 5-hydroxyisourate (5-HIU) hydrolase (C-terminal domain). TTL is a member of the transthyretin-related protein family (TRP), which comprises a number of proteins with sequence homology to transthyretin (TTR) and the characteristic C-terminal sequence motif Tyr-Arg-Gly-Ser. TRPs are single domain proteins that form tetrameric structures with 5-HIU hydrolase activity. Experimental evidence is fundamental for knowing if TTL is a tetrameric protein, formed by the association of the 5-HIU hydrolase domains and, in this case, if the structural arrangement allows for OHCU decarboxylase activity. This work reports about the biochemical and functional characterization of TTL.

**Results:**

The TTL gene was cloned and the protein expressed and purified for biochemical and functional characterization. The results show that TTL is composed of four subunits, with a moderately elongated shape. We also found evidence for 5-HIU hydrolase and OHCU decarboxylase activities *in vitro*, in the full-length protein.

**Conclusions:**

The *Arabidopsis thaliana *transthyretin-like (TTL) protein is a tetrameric bifunctional enzyme, since it has 5-HIU hydrolase and OHCU decarboxylase activities, which were simultaneously observed *in vitro*.

## Background

The *Arabidopsis thaliana *transthyretin-like protein (TTL) was first identified as a potential substrate of Brassinosteroid-Insensitive 1 (BRI1), the principal brassinosteroid (BR) receptor, playing a negative role in BR-mediated plant growth [1].

Sequence analysis shows that TTL displays an N-terminal domain corresponding to 2-oxo-4-hydroxy-4-carboxy-5-ureidoimidazoline (OHCU) decarboxylase, and a C-terminal domain that has approximately 42% sequence identity with transthyretin (TTR), a vertebrate-specific transport protein [1,2]. TTR is a plasma protein which transports the thyroid hormones 3,5,3',5'-tetraiodo-L-thyronin (T_4_) and 3,5,3-triiodo-L-thyronin (T_3_) as well as retinol by association with the retinol-binding protein [3]. TTRs are homotetrameric proteins, each monomer containing two four-stranded β-sheets and one short α-helix. They have a channel that runs through the dimer-dimer interface, where two identical thyroid hormone binding sites are located [4,5]. Proteins with sequence homology to TTR and with the characteristic C-terminal sequence motif Tyr-Arg-Gly-Ser, which is absent in TTRs, are named transthyretin-related proteins (TRPs) and are found in bacteria, fungi, animals (both vertebrate and invertebrate) and plants [2,6,7]. TRPs are thought to be TTR ancestors [2,7].

Three TTL splice variants were reported; two of them are cytoplasmic (311- and 286-residues each) and the other one is peroxisomal (324-residues) [8]. In all three variants the N-terminal domain, OHCU decarboxylase, is composed of 180 amino acids, whereas the C-terminal domain varies in length, having 144, 131 and 106 amino acids for the 324-, 311- and 286-residues isoforms, respectively [2,8]. The 324-residues isoform contains a type-2 peroxisomal targeting sequence (PTS-2), which is deleted in the other two isoforms [2,8]. In most eukaryotes, 5-HIU hydrolases contain a PTS-2 and OHCU decarboxylases contain a type-1 peroxisomal targeting sequence (PTS-1), except in plants [9]. In *A. thaliana *these two enzymes are fused into a single polypeptide chain, containing a PTS-2 sequence, between the two domains, in the 324-residues isoform.

In a previous study, the C-terminal domain of TTL was expressed and studied separately. This single-domain was named transthyretin-like protein (TLP) [2] and is distinct from TTL, which contains both this C-terminal domain, with 5-HIU hydrolase activity, and a N-terminal domain, with OHCU decarboxylase activity, fused into the same polypeptide chain [8]. TLP was described as a tetramer [2]. The three-dimensional structure of TRPs from *Salmonella Dublin, Escherichia coli*, zebrafish and the homologous domain from *Bacillus subtilis *were determined [10-13]. The overall structures of these TRPs and also the model predicted for TLP [2] are very similar to those of TTRs, as expected due to sequence homology, although functionally they are different proteins. TRPs are hydrolases with a role in uric acid degradation [7,14] and they do not bind thyroid hormones [2,6]. TTL is the *A. thaliana *TRP member, which contains an extra domain of 180 amino acids as compared to other TRPs. Until now, only TRPs from *Magnetospirillum magnetotacticum*, *Bradyrhizobium japonicum*,*Bacillus subtilis *and plant species have been described as composed of two sequence domains, one related to TTR and the other with features common to OHCU decarboxylases [2,7,9].

**Figure 1:**
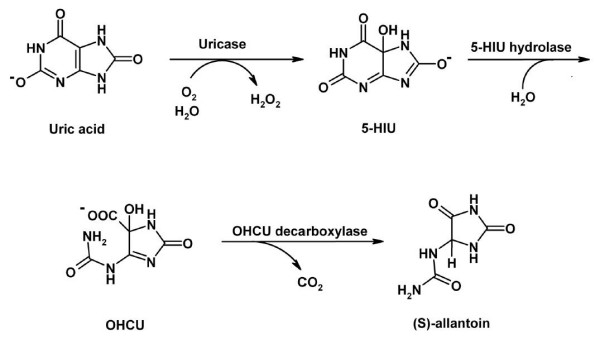
**The uric acid degradation pathway**.

TTL was predicted by sequence homology as being a bifunctional enzyme with 5-hydroxyisourate (5-HIU) hydrolase and OHCU decarboxylase activities, catalysing the two final steps in the uric acid degradation pathway (Fig. 1) [8]. In most organisms, uric acid, the end product of purine degradation, is catabolised to allantoin. The process is initiated by uricase, which oxidizes uric acid into 5-HIU; this intermediate compound is then hydrolysed by 5-HIU hydrolase, leading to OHCU. Finally, a third enzyme, OHCU decarboxylase, catalyses the decarboxylation of OHCU producing (*S*)-allantoin [9]. The spontaneous degradation of uric acid follows the same pathway, resulting into 5-HIU and OHCU intermediates, which can be further oxidized, producing potentially harmful reactive species. However, many organisms have an enzymatic pathway, possibly to convert these intermediates more rapidly into allantoin, a much less reactive species. The spontaneous pathway produces racemic allantoin, whereas the enzymatic pathway produces exclusively (*S*)-allantoin [9].

So far, two different functions have been assigned to TTL: i) a signalling role to the cytoplasmic isoforms and ii) an enzymatic role to the peroxisomal isoform. While the signalling role has already been studied [1], it is not known if the two enzymatic sequence domains that are in this protein fused into a long polypeptide chain do not compromise the role of each domain in an isolated form. In this work we studied the 311-residue cytoplasmic isoform, referred as TTL throughout the manuscript, to address the protein enzymatic role.

## Results

### TTL oligomerization state

**Figure 2:**
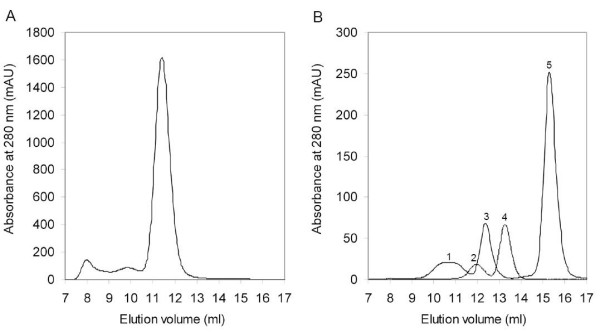
**Analytical gel filtration chromatograms for determination of TTL Stokes radius**. (A) shows a chromatogram of recombinant TTL (elution volume: 11.4 ml). In (B), two chromatograms of standard Stokes radius markers are superposed, where 1 refers to ferritin (Stokes radius: 6.1 nm; elution volume: 10.7 ml), 2 to aldolase (4.81 nm; 12.0 ml), 3 to albumin (3.55 nm; 12.4 ml), 4 to ovalbumin (3.05 nm; 13.3 ml) and 5 to chymotrypsinogen A (2.09 nm; 15.3 ml).

Recombinant TTL was purified and used to investigate the protein oligomerization state. We decided to use the method developed by Siegel and Monty in 1966 [15], because it allows calculation of the molecular mass of a protein independently of its shape, using a combination of the Stokes radius (*a*) derived from gel filtration chromatography and the sedimentation coefficient (S) obtained from density gradient centrifugation. The gel filtration chromatograms are presented in Fig. 2, showing in Fig. 2A a major peak of oligomeric TTL and in Fig. 2B superposed chromatograms of standard Stokes radius markers. Using the equations described in Methods, section on "Determination of the protein oligomeric state", *a *and S were estimated to be 5.3 nm and 6.3 S, respectively, and with these values the native molecular mass for TTL was estimated to be 137.3 kDa. Since each TTL subunit has 36.6 kDa, we concluded that TTL is a tetramer, which is in agreement with the fact that TTR, as well as *A.thaliana *TLP, also have a tetrameric structure [2]. Moreover, the calculated frictional ratio, 1.5, is consistent with a moderately elongated non-globular protein [15].

### Thyroxin (T_4_)-binding assays

**Figure 3:**
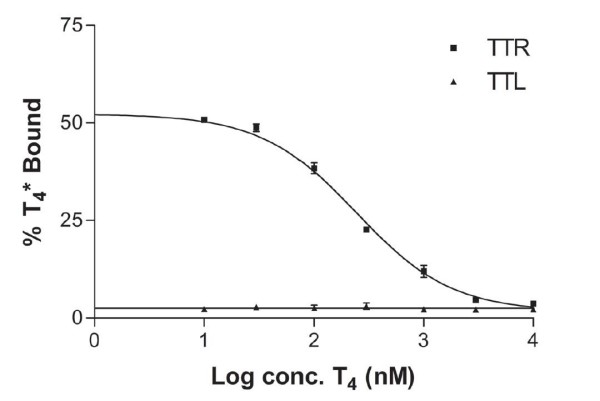
**T_4_-binding assay**. The percentage of binding of radioactive T_4 _* was gradually reduced by increasing the concentration of T_4 _in the TTR solution, but not in TTL. Each curve is representative of two independent measurements and standard deviations are indicated by error bars.

Since TTR is a tetramer with two equivalent T_4_-binding sites, we decided to study the binding of T_4 _to TTL, using radiolabeled thyroxin and TTR as the sample control (Fig. 3). The TTR control has the typical behaviour of a specific T_4_-binding protein, in which the increase of cold T_4 _concentration causes the displacement of specifically bound radiolabeled T_4 _*. On the contrary, T_4 _*-binding to TTL, besides being very low, was constant at different T_4 _concentrations, showing a non-specific binding. This is a very different behaviour as compared to TTR, for which non-specific binding was only detected at the highest T_4 _concentration. We concluded that the binding of T_4 _to TTL is not significant and clearly non-specific, since it is not altered in the presence of a competitor.

**Figure 4:**
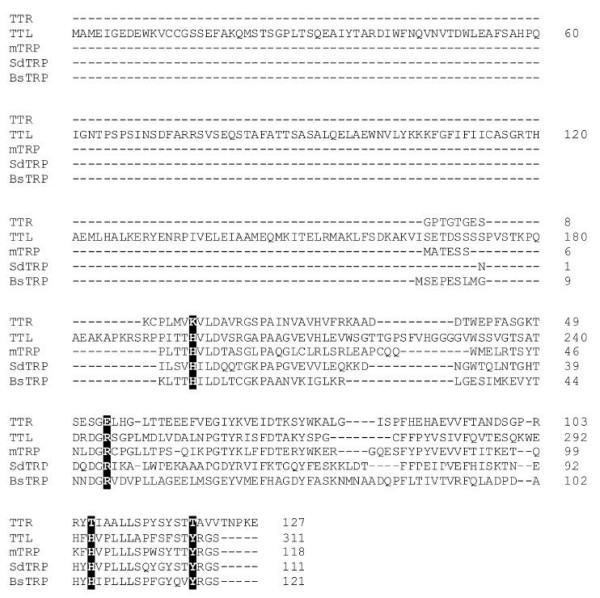
**Sequence alignment of human TTR, TTL and TRPs from mouse (mTRP), *S. dublin *(SdTRP) and *B. subtilis *(BsTRP)**. Amino acids involved in the binding of T_4 _to TTR and their substitutes in TTL and TRPs are presented in black. Sequence alignment was obtained using *ClustalW *[24].

Sequence alignment of human TTR, TTL and TRPs from *Salmonella dublin*, *B. subtilis *and mouse are presented in Fig. 4. As mentioned previously, only the C-terminal domain of TTL has homology with TTR or TRPs. Several amino acids were reported as important for the binding of T_4 _to TTR, namely Lys15, Glu54, Thr106 and Thr119 [5]. Interestingly, they are not conserved in TTL, being replaced by His196, Arg245, His295 and Tyr308, respectively (Fig. 4), which are amino acids conserved in TRPs, and were reported to be important for 5-HIU hydrolase catalytic activity [10,13,16]. According to our sequence alignment data (Fig. 4) and the three-dimensional structure of several TTR and TRP proteins [5,10-13], these substitutions will probably have two main effects: i) create a narrower region at the inner part of the binding site due to the replacement of Thr for Tyr, which has a larger side chain and ii) alter the surface topography and introduce charge differences at the entrance of the channel, due to substitutions Lys-His, Glu-Arg and Thr-His. These modifications will probably exclude the binding of T_4 _to TRPs and TTL.

### TTL enzymatic activities

**Figure 5:**
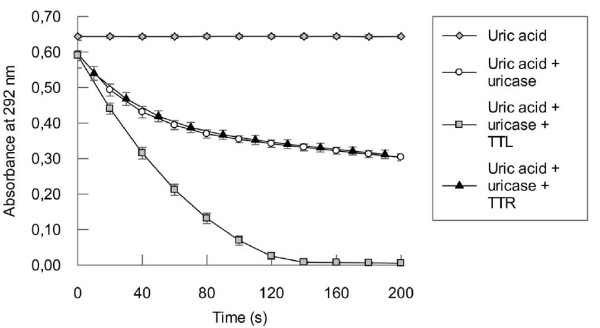
**Uricase activity is accelerated by TTL**. The enzymatic oxidation of uric acid was monitored by decrease in absorbance at 292 nm. Uricase was pre-incubated with TTL or TTR for 5 minutes at 22°C and then uric acid was added to start the reaction. TTR was used as negative control.

Since TRPs are functionally associated to 5-HIU hydrolases, we decided to study the predicted 5-HIU hydrolase and OHCU decarboxylase activities for TTL *in vitro*. The experiments were started by testing how TTL would affect the activity of uricase over uric acid by monitoring the differences in absorbance at 292 nm in the absence or presence of TTL (Fig. 5). Although uricase alone was able to decompose uric acid, its activity was significantly accelerated in the presence of TTL. By contrast, TTR did not influence this reaction. This result is consistent with the predicted 5-HIU hydrolase and OHCU decarboxylase activities. The rapid consumption of 5-HIU and OHCU should favour an equilibrium shift in the reaction catalysed by uricase.

**Figure 6:**
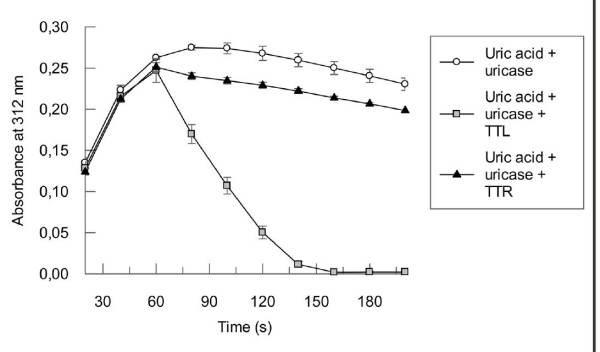
**5-HIU hydrolase activity of TTL**. Uricase was pre-incubated with uric acid to produce 5-HIU. When the absorbance measured at 312 nm reached its maximum (in approximately 60 seconds), TTL or TTR were added. The observed absorbance decrease was monitored for the degradation of 5-HIU, which was complete in 100 seconds (from 60 to 160 seconds).

5-HIU hydrolase activity was demonstrated *in vitro *for single-domain TRPs present in *S. dublin*, *B. subtilis *and mouse [10,14,16]. The authors reported that uric acid was converted into 5-HIU by uricase and both the formation and subsequent degradation of this compound was monitored at 312 nm, since OHCU does not absorb at this wavelength [17]. In our assay conditions, within 60 seconds incubation of uric acid with the *Candida sp*. uricase, the production of 5-HIU reached the maximum level (Fig. 6). At this point, TTL was added to the reaction solution resulting in a rapid decrease in absorbance at 312 nm (Fig. 6). As expected, addition of TTR, which was used as the negative control, failed to accelerate 5-HIU degradation. This result shows that TTL is an enzyme that facilitates the hydrolysis of 5-HIU and also indicates that most probably four protein C-terminal domains associate forming a tetrameric structure, since the 5-HIU hydrolase catalytic site are located at the dimer-dimer interface in all structurally and functionally characterised TRP members [10,13].

**Figure 7:**
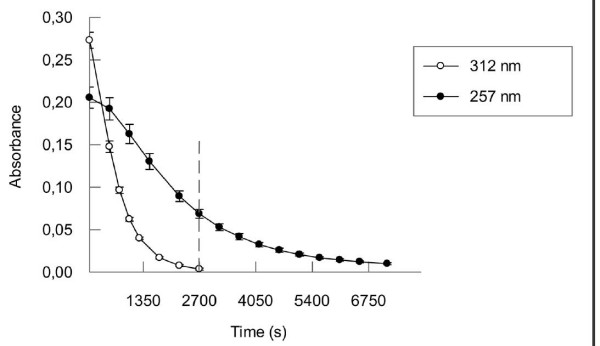
**Spontaneous degradation of 5-HIU and OHCU**. Uricase was pre-incubated with uric acid during 60 seconds to produce 5-HIU (data not shown) and degradation was followed at 312 nm. The degradation of both 5-HIU and OHCU was followed at 257 nm. Since spontaneous degradation of 5-HIU is complete in 2640 seconds (from 60 to 2700 seconds), as verified at 312 nm, after this point (marked with dash line), absorbance curve at 257 nm is exclusively due to the spontaneous degradation of OHCU. (X axis starts at 60 s).

Although the crystal structure of the N-terminal OHCU domain of TTL from *A. thaliana*, which contains conserved residues critical for the OHCU decarboxylase activity, was recently reported [18], its predicted enzyme activity was not experimentally demonstrated. In fact, it was only observed *in vitro *in an isolated domain of a bifunctional enzyme from *B. subtilis *and also in zebrafish OHCU decarboxylase [18,19]. Monitoring the decarboxylase activity of TTL by spectrophotometry is not straightforward since OHCU absorbs below 300 nm and its absorption spectrum overlaps with that of 5-HIU [17]. Therefore, measurements at 257 nm are likely to be a sum of contributions by 5-HIU and OHCU. However, the spontaneous degradation observed at this wavelength is clearly distinct from what is observed at 312 nm (Fig. 7). The spontaneous degradation followed at 312 nm shows that 5-HIU is completely degraded in 2640 seconds (from 60 s to 2700 s Fig. 7), thus the spontaneous absorbance decay measured after this point at 257 nm (marked with dash line in Fig. 7) is exclusively due to the spontaneous degradation of OHCU. Therefore this degradation was used as control in the experiments testing the effect of TTL on degradation of OHCU.

**Figure 8:**
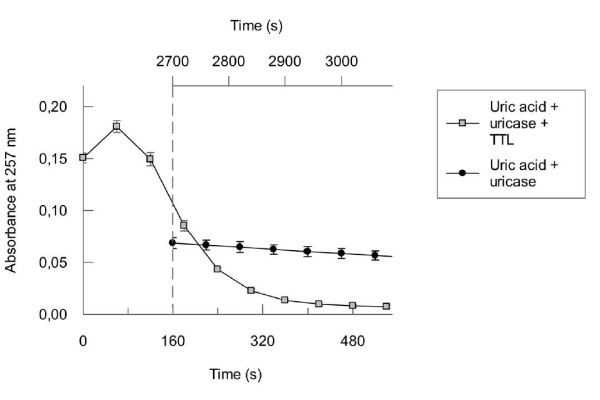
**OHCU decarboxylase activity of TTL**. Uricase was pre-incubated with uric acid to produce 5-HIU. When the absorbance measured at 257 nm reached its maximum (in approximately 60 seconds), TTL was added. Since in presence of TTL, 5-HIU is completely degraded in 100 seconds (from 60 to 160 seconds; see Fig. 6), the absorbance decay after this point (marked with a dash line) measured at 257 nm is only due to OHCU degradation. The equivalent control for the spontaneous degradation of OHCU was built from Fig. 7 (in this case the time was marked in the upper X axis). Starting from the dashed line, both X axes show the same time period of 390 seconds (160 to 550 seconds and 2700 to 3090 seconds).

In presence of TTL the degradation of 5-HIU followed at 312 nm was completed in 100 seconds (from 60 s to 160 s in Fig. 6) therefore after this point the absorbance decay measured at 257 nm (marked with a dash line in Fig. 8) corresponds to the degradation of OHCU affected by TTL. The rate of this degradation is excessively high as compared to the control spontaneous degradation of OHCU (Fig. 8). Addition of TTL accelerated significantly the degradation of OHCU, supporting the decarboxylase activity of the protein.

Taken together, our results demonstrated that TTL has 5-HIU hydrolase and OHCU decarboxylase activities *in vitro*, and they are both performed at the same time.

## Discussion

It seems that the TTR domain evolved from an enzyme present in bacteria, fungi, plants and animals to a hormone transporter present in vertebrates [2,7,12]. Its function changed completely, although there are only minor differences in sequence. In the present study, we showed that TTL from *A. thaliana *is a tetramer with a moderately elongated shape. The tetrameric structure is in agreement with the observation that the protein has 5-HIU hydrolase activity. We expect that four C-terminal domains associate in a tetramer forming two active sites, as reported for TRPs, which contain only one domain. Moreover, the elongated shape is possibly due to the N-terminal OHCU decarboxylase domain associated to each of the four C-terminal sequences forming the tetramer. The molecular model of this domain was determined in the presence of allantoin [18]; it is an alpha-domain that associates as dimers in its crystal form. However, a careful inspection of the available molecular models for TRPs and OHCU decarboxylase domain indicates that allantoin binding site is not in the region of the interaction between the two subunits that form the dimer [18] and this kind of association most probably will not be present in TTL, since the TTL tetramer will be formed through contacts among the C-terminal domains.

We also demonstrated that TTL does not bind T_4 _*in vitro*, corroborating the observation that the ligand-binding site has characteristics which are different from those present in TTR. These results are in agreement with previous observations, since the functionally characterized TRPs do not bind thyroid hormones [2,6] and are 5-HIU hydrolases with active sites located in the regions corresponding to the T_4_-binding sites in TTR [7,10,12,13]. The OHCU decarboxylase activity was also detected in the full-length TTL, indicating that the N-terminal domains do not inhibit the active sites formed by the C-terminal regions; the reverse is also valid, thus supporting the idea of a bifunctional enzyme.

TTL was initially characterized as a specific BRI1-interactor that regulates BR-mediated plant growth [1]. Here we show that the same protein is also enzymatically active. Several proteins with at least two totally different functions have been reported and named moonlighting proteins; the switch between functions is due to changes in cellular localization, cell type, oligomeric state or cellular concentration of a ligand [20,21]. We think that TTL is a moonlighting protein that switches its function according to its subcellular location. In eukaryotes, peroxisomes are the major organelles where oxidative reactions with molecular oxygen consumption are carried out. In these organisms, uricase is located in peroxisomes, oxidizing uric acid to 5-HIU [9]. As a consequence, 5-HIU accumulates in peroxisomes, providing the initial TTL substrate. Therefore, in peroxisomes, where uric acid degradation occurs, TTL, through its 324-residues isoform, functions as a bifunctional enzyme with 5-HIU hydrolase and OHCU decarboxylase activities. In the cytoplasm, 5-HIU and OHCU are absent, since they are produced in peroxisomes. Consequently, the TTL 311-residues isoform most probably will not have these enzymatic functions. Moreover, it was shown to interact with BRI1, mediating BR-regulated plant growth. BRI1 is an intrinsic membrane protein, located in the plasma membrane and containing a cytoplasmic kinase domain that interacts with TTL [1]. The main difference between the 324- and 311-residues isoforms is the presence or absence of PTS-2. The subcellular location of three TTL isoforms is probably fundamental to define their role *in vivo*. TTL 324-residues isoform location into peroxisomes was already shown by fluorescence microscopy, and provided conclusive results regarding its import [8]. It was also observed that its import efficiency was low and it was hypothesized that could result from a low accessibility of the internal targeting signal to the peroxisomal import machinery. Internal PTS-2 signal sequences are thought to be rare in nature and to occur preferentially in bifuctional enzymes resulting from gene fusion [8]. The 311-residues isoform will probably remain in the cytoplasm *in vivo*, since it does not contain the PTS-2 sequence [1,8]. Therefore in the absence of uric acid degradation intermediates, it interacts with the plasma membrane-located BRI1, mediating BR-regulated plant growth [1].

Attempts for the three-dimensional structure determination of TTL are in progress and should represent a fundamental step in order to clarify TTL function, namely its signalling role in BR-mediated responses.

## Conclusions

The aminoacid sequence of TTL from *A. thaliana *reveals two domains similar to 5-HIU hydrolase and OHCU decarboxylase. As a consequence, TTL was predicted to be a bifunctional enzyme. Here we reported the first experimental data showing that in fact the protein has both enzymatic activities *in vitro*. Moreover, we confirmed that TTL is in fact a bifunctional enzyme, by showing that it performs both activities simultaneously.

## Methods

### Cloning, expression and purification of A. *thaliana *TTL

TTL ORF was amplified by PCR and cloned into pET-28a(+) (Novagen). Recombinant TTL was overexpressed in *E. coli *BL21 (DE3) codon plus and purified as follows: cells were grown in LB at 37°C to OD_600 _= 0.8 and expression induced with 1 mM IPTG for 18 hours at 20°C. Cells were harvested (10500 g, 20 minutes), ressuspended in 140 mM NaCl, 2.7 mM KCl, 1.0 mM KH_2_PO_4_, 50 μg/ml lysozyme, 1 mM phenylmethylsulfonyl fluoride (PMSF), 1 μg/ml DNase I, 100 μM MgCl_2_, 10 mM Na_2_HPO_4_, pH 7.4 and lysed by sonication. Lysates were centrifuged (27200 g, 20 minutes), supernatants filtered and loaded onto a 5 ml HisTrap HP column (GE Healthcare) previously equilibrated with buffer A (20 mM imidazole, 500 mM NaCl, 20 mM NaH_2_PO_4_/Na_2_HPO_4_, pH 7.4). After a washing step with 20% buffer B (500 mM imidazole, 500 mM NaCl, 20 mM NaH_2_PO_4_/Na_2_HPO_4_, pH 7.4), TTL was eluted with 50% buffer B. Fractions containing His-tagged TTL were dialyzed against buffer A followed by buffer C (150 mM NaCl, 1 M EDTA, 20 mM Tris-HCl, pH 8.0), concentrated and loaded onto a HiPreP 26/60 Sephacryl S-300 HR column (GE Healthcare). After an isocratic elution in buffer C, TTL containing fractions were pooled and concentrated to 7 mg/ml. Protein concentration was determined by absorption at 280 nm using a theoretical 49430 M^-1^cm^-1 ^molar extinction coefficient [22].

### Determination of the protein oligomeric state

For analytical size-exclusion chromatography a Superose 12 10/300 column (GE Healthcare) was equilibrated with buffer C at 0.5 ml/min and calibrated with protein standards of known Stokes radius. The Stokes radius (*a*) for the experimental data was calculated using: (-logK_av_)^1/2 ^= f(*a*). For isokinetic rate zonal ultracentrifugation, two continuous gradients of 5 to 20% sucrose (Merck) were prepared in buffer C, calibrated with standard proteins of known sedimentation coefficient and ran for 25 hours and 15 minutes at 260800 g in a Beckman SW41 Ti rotor. TTL native molecular mass (M) and frictional ratio (*f*/*f*_0_) were calculated according to Siegel and Monty [15], using the following equations:

where M represents the molecular mass, *a *the Stokes radius, s the sedimentation coefficient, v the partial specific volume, *f*/*f*_0 _the frictional ratio, η the viscosity of the medium, ρ the density of the medium and N Avogadro's constant. Partial specific volume for TTL was calculated from the amino acid sequence of the protein using the program SEDNTERP v1.08 http://www.jphilo.mailway.com/default.htm.

### Thyroxin-binding assays

The assays were performed as previously described [23], using 100 nM TTR or TTL, 5-10 × 10^4 ^counts per minute (cpm) radiolabeled thyroxin, ^125^I-T_4 _(T_4 _*) (Perkin Elmer) and different cold T_4_(Sigma) concentrations. Counts were measured in a Wallac 1470 wizard™ automatic gamma counter.

### Enzyme assays

The enzymatic assays were based on previously described procedures for enzymes with sequence similarity to the TTL hydrolase domain [10,14]. Briefly, 500 μl assay mixtures contained 0.05 units/ml uricase from *Candida sp*. (Sigma) in 50 mM potassium phosphate buffer pH 7.8. When 50 μM uric acid (Sigma) were added, 5-HIU and OHCU were generated *in situ *and their degradation was followed at 312 nm and 257 nm, in the presence and absence of 0.003 μM TTL or TTR. TTL effect on uricase activity was monitored at 292 nm in the same conditions. Triplicate measurements were done aerobically at 22°C.

## Abbreviations

5-HIU: 5-hydroxyisourate; BR: Brassinosteroid; BRI1: Brassinosteroid-Insensitive 1; OHCU: 2-oxo-4-hydroxy-4-carboxy-5-ureidoimidazoline; PTS-2: type-2 peroxisomal targeting sequence; T_4_: 3,5,3',5'-tetraiodo-L-thyronin, thyroxin; T_3_: 3,5,3-triiodo-L-thyronin; T_4 _*: radiolabeled thyroxin; TLP: transthyretin-like potein; TTL: transthyretin-like protein; TRP: transthyretin-related protein; TTR: transthyretin.

## Authors' contributions

JP carried out the biochemical and functional analysis work under ZS and FFS's supervision; SM collaborated on the protein expression work; MRA supervised the T_4 _binding studies; JL supplied the TTL-coding vector and discussed the results; AMD coordinated the study. All authors gave ideas, revised, read and approved the final manuscript.

## References

[B1] Nam KE Li J The Arabidopsis transthyretin-like protein is a potential substrate of Brassinosteroid Insensitive 1 The Plant Cell 2004 16 2406 2417 10.1105/tpc.104.023903 15319482 PMC520942

[B2] Hennebry SC Wright HM Likic VA Richardson SJ Structural and functional evolution of transthyretin and transthyretin-like proteins Proteins: Struct Func Bioinfo 2006 64 1024 1045 10.1002/prot.21033 16783790

[B3] Power DM Elias NP Richardson SJ Mendes J Soares CM Santos CRA Evolution of the thyroid hormone-binding protein, transthyretin Gen Comp Endocrinol 2000 119 241 255 10.1006/gcen.2000.7520 11017772

[B4] Blake CC Geisow MJ Oatley SJ Rérat B Rérat C Structure of prealbumin: secondary, tertiary and quaternary interactions determined by Fourier refinement at 1.8 Å J Mol Biol 1978 121 339 356 10.1016/0022-2836(78)90368-6 671542

[B5] Wojtczak A Cody V Luft JR Pangborn W Structures of human transthyretin complexed with thyroxine at 2.0 Å resolution and 3',5'-dinitro-N-acetyl-L-thyroxine at 2.2 Å resolution Acta Crystallogr 1996 D52 758 765 10.1107/S0907444996003046 15299640

[B6] Eneqvist T Lundberg E Nilsson L Abagyan R Sauer-Eriksson AE The transthyretin-related protein family Eur J Biochem 2003 270 518 532 10.1046/j.1432-1033.2003.03408.x 12542701

[B7] Hennebry SC Evolutionary changes to transthyretin: structure and function of a transthyretin-like ancestral protein FEBS J 2009 276 5367 5379 10.1111/j.1742-4658.2009.07246.x 19725880

[B8] Reumann S Babujee L Ma C Wienkoop S Siemsen T Antonicelli GE Rasche N Lüder F Weckwerth W Jahn O Proteome analysis of *Arabidopsis *leaf peroxisomes reveals novel targeting peptides, metabolic pathways, and defense mechanisms The Plant Cell 2007 19 3170 3193 10.1105/tpc.107.050989 17951448 PMC2174697

[B9] Ramazzina I Folli C Secchi A Berni R Percudani R Completing the uric acid degradation pathway through phylogenetic comparison of whole genomes Nat Chem Biol 2006 2 144 148 10.1038/nchembio768 16462750

[B10] Hennebry SC Law RHP Richardson SJ Buckle AM Whisstock JC The crystal structure of the transthyretin-like protein from *Salmonella dublin*, a prokaryote 5-hydroxyisourate hydrolase J Mol Biol 2006 359 1389 1399 10.1016/j.jmb.2006.04.057 16787778

[B11] Lundberg E Backstrom S Sauer UH Sauer Eriksson AE The transthyretin-related protein: Structural investigation of a novel protein family J Struct Biol 2006 155 445 457 10.1016/j.jsb.2006.04.002 16723258

[B12] Zanotti G Cendron L Ramazzina I Folli C Percudani R Berni R Structure of zebra fish HIUase: insights into evolution of an enzyme to a hormone transporter J Mol Biol 2006 363 1 9 10.1016/j.jmb.2006.07.079 16952372

[B13] Jung D-K Lee Y Park SG Park BC Kim G-H Rhee D Structural and functional analysis of PucM, a hydrolase in the ureide pathway and a member of the transthyretin-related protein family Proc Nat Acad Sci USA 2006 103 9790 9795 10.1073/pnas.0600523103 16782815 PMC1502532

[B14] Lee Y Lee DH Kho CW Lee AY Jang M Cho S Lee CH Lee JS Myung PK Park BC Park SG Transthyretin-related proteins function to facilitate the hydrolysis of 5-hydroxyisourate, the end product of the uricase reaction FEBS Lett 2005 579 4769 4774 10.1016/j.febslet.2005.07.056 16098976

[B15] Siegel L Monty K Determination of molecular weights and frictional ratios of proteins in impure systems by use of gel filtration and density gradient centrifugation. Application to crude preparations of sulfite and hydroxylamine reductases Biochim Biophys Acta 1966 112 346 362 10.1016/0926-6585(66)90333-5 5329026

[B16] Lee Y Park BC Lee DH Bae K-H Cho S Lee CH Lee JS Myung PK Park SG Mouse transthyretin-related protein is a hydrolase which degrades 5-hydroxyisourate, the end product of the uricase reaction Mol Cells 2006 22 141 145 17085964

[B17] Kahn K Tipton PA Spectroscopic characterization of intermediates in the urate oxidase reaction Biochemistry 1998 37 11651 11659 10.1021/bi980446g 9709003

[B18] Kim K Park J Rhee S Structural and functional basis for (*S*)-allantoin formation in the ureide pathway J Biol Chem 2007 282 23457 23464 10.1074/jbc.M703211200 17567580

[B19] Cendron L Berni R Folli C Ramazzina I Percudani R Zanotti G The structure of 2-oxo-4-hydroxy-4-carboxy-5-ureidoimidazoline decarboxylase provides Insights into the mechanism of uric acid degradation J Biol Chem 2007 282 18182 18189 10.1074/jbc.M701297200 17428786

[B20] Jeffery CJ Moonlighting proteins Trends Biochem Sci 1999 24 8 11 10.1016/S0968-0004(98)01335-8 10087914

[B21] Jeffery CJ Moonlighting proteins: old proteins learning new tricks Trends Genetics 2003 19 415 417 10.1016/S0168-9525(03)00167-7 12902157

[B22] Pace CN Vajdos F Fee L Grimsley G Gray T How to measure and predict the molar absorption coefficient of a protein Prot Science 1995 4 2411 2423 10.1002/pro.5560041120 PMC2143013 8563639

[B23] Almeida MR Damas AM Lans MC Brouwer A Saraiva MJ Thyroxine binding to transthyretin Met 119. Comparative studies of different heterozygotic carriers and structural analysis Endocrine 1997 6 309 315 10.1007/BF02820508 9368688

[B24] Thompson JD Higgins DG Gibson TJ CLUSTAL W: improving the sensitivity of progressive multiple sequence alignment through sequence weighting, position-specific gap penalties and weight matrix choice Nucleic Acids Res 1994 22 4673 4680 10.1093/nar/22.22.4673 7984417 PMC308517

